# Quercetin-induced cardioprotection against doxorubicin cytotoxicity

**DOI:** 10.1186/1423-0127-20-95

**Published:** 2013-12-20

**Authors:** Jing-Yi Chen, Ren-Yu Hu, Hsiu-Chuan Chou

**Affiliations:** 1 Department of Applied Science, National Hsinchu University of Education, Hsinchu, Taiwan

**Keywords:** Quercetin, Doxorubicin, Proteomics, DIGE, MALDI-TOF, Cardiomyocytes

## Abstract

**Background:**

Cancer has continually been the leading cause of death worldwide for decades. Thus, scientists have actively devoted themselves to studying cancer therapeutics. Doxorubicin is an efficient drug used in cancer therapy, but also produces reactive oxygen species (ROS) that induce severe cytotoxicity against heart cells. Quercetin, a plant-derived flavonoid, has been proven to contain potent antioxidant and anti-inflammatory properties. Thus, this *in vitro* study investigated whether quercetin can decrease doxorubicin-induced cytotoxicity and promote cell repair systems in cardiomyocyte H9C2 cells.

**Results:**

Proteomic analysis and a cell biology assay were performed to investigate the quercetin-induced responses. Our data demonstrated that quercetin treatment protects the cardiomyocytes in a doxorubicin-induced heart damage model. Quercetin significantly facilitated cell survival by inhibiting cell apoptosis and maintaining cell morphology by rearranging the cytoskeleton. Additionally, 2D-DIGE combined with MALDI-TOF MS analysis indicated that quercetin might stimulate cardiomyocytes to repair damage after treating doxorubicin by modulating metabolic activation, protein folding and cytoskeleton rearrangement.

**Conclusion:**

Based on a review of the literature, this study is the first to report detailed protective mechanisms for the action of quercetin against doxorubicin-induced cardiomyocyte toxicity based on in-depth cell biology and proteomic analysis.

## Background

Doxorubicin is a chemotherapy drug, commonly used in various cancer treatments, such as breast cancer, lung cancer and several other carcinoma types [1-3]. The principal mechanism of doxorubicin is chelating DNA, inhibiting topoisomerase II and then producing free radicals to kill cancer cells. Reported side effects of doxorubicin include cardiotoxicity, comprising cardiomyopathy and ultimately fatal congestive heart failure. Because myocardia are particularly sensitive to reactive oxygen species (ROS), cumulative doxorubicin *in vivo* causes irreversible damage to heart cells, thus restricting clinical use of this drug [4]. Although the specific causal mechanism of doxorubicin-induced cardiotoxicity remains largely unclear, most of the evidence has indicated that doxorubicin is reduced to its semiquinone form by a mitochondria electron transport system. The semiquinone subsequently reacts with oxygen, iron, and hydrogen peroxide to produce ROS causing cell apoptosis and myocyte damage [5,6]. In addition, global analysis of doxorubicin-induced cellular oxidative stress has indicated that doxorubicin treatment contributes to the over-expression of anti-oxidant proteins such as glutathione reductase and peroxiredoxin in brain cells, lung cells and heart cells [7-9].

Quercetin, a type of polyphenolic compound found in various plant products, possesses anti-oxidant, anti-proliferative, anti-inflammatory and anti-histamine properties. Several reports have indicated that quercetin exerts protective effects on various cells, including myocytes, testes, renal cells and liver cells in ischemia and reperfusion injury [10]. A study conducted in 1992 determined that quercetin reduces the oxidative stress caused by ischemia and reperfusion in cardiomyocytes by inhibiting the xanthine dehydrogenase and xanthine oxidase system [11]. Several reports have also indicated that quercetin and isorhamnetin scavenge ROS and inhibit the activation of ERK and MAP kinase in ROS-induced cardiomyopathy [12,13]. In cancer therapy, combining quercetin with doxorubicin augmented the effects of doxorubicin in highly invasive breast cancer cells [14] and can protect cardiomyocytes from doxorubicin-induced toxicity by chelating iron, inducing antioxidant activity, and inhibiting carbonyl reductase [15]. Regarding proteomic analysis, the results also indicated that quercetin could down-regulate Ras GTPase-activating-like proteins and heat shock protein-90 to reduce cell migratory ability and cell survival, respectively, in malignant cancers [16,17]. Although quercetin has been reported to play a role in protecting myocardial cells from ischemia and reperfusion injury, its protective mechanism remains unclear.

To investigate the role of quercetin in alleviating doxorubicin-induced cardiotoxicity, we examined the protective ability of quercetin in doxorubicin-treated rat cardiomyocytes by performing cell biological assays, such as cell viability and apoptotic analysis, as well as a quantitative proteomic analysis based on 2D-DIGE and MALDI-TOF MS identification [18].

## Methods

### Chemicals and reagents

Generic chemicals were purchased from Sigma-Aldrich (St. Louis, USA), while reagents for 2D-DIGE were purchased from GE Healthcare (Uppsala, Sweden). All primary antibodies were purchased from Genetex (Hsinchu, Taiwan) and anti-mouse, and anti-rabbit secondary antibodies were purchased from GE Healthcare (Uppsala, Sweden). All the chemicals and biochemicals used in this study were of analytical grade.

### Cell lines and cell culture

The rat cardiomyocyte cell line H9C2 was purchased from American Type Culture Collection (ATCC) (Manassas, VA) and was maintained in Dulbecco’s modified Eagle’s medium (DMEM) supplemented with 10% (v/v) FCS, L-glutamine (2 mM), streptomycin (100 μg/mL) and penicillin (100 IU/mL) (all from Gibco-Invitrogen Corp., UK). Cells were incubated in a humidified incubator at 37°C and 5% CO_2_. and passaged at 80-90% confluence by trypsinization according to standard procedures.

### MTT cell viability assay

The detailed MTT experimental procedure has been described in our previous study [19].

### Immunofluorescence

Cells were plated onto coverslips (VWR international) for overnight incubation and subsequently fixed with PBS containing 4% (v/v) paraformaldehyde for 25 min. After washing three times in PBS, samples were permeabilized in PBS containing 0.2% (v/v) Triton X-100 for 10 min. and then rinsed and blocked in PBS containing 5% (w/v) BSA for 10 min prior to incubate with primary antibodies diluted in 2.5% BSA/PBS for 1 h. After PBS washings, samples were incubated with the appropriate fluorescently labeled secondary antibodies diluted in 2.5% BSA/PBS for 1 h. Samples were then washed three times with PBS and briefly rinsed with ddH_2_O twice before applying to Vectashield mounting medium (Vector Lab). Coverslip edges were sealed with nail polish onto glass slides (BDH) and then air-dried in the dark at 4°C. For image analysis, cells were visualized using a Zeiss Axiovert Z1 fluorescent microscope (Carl Zeiss Inc., Germany). Identical laser intensities were used to detect the same immunostained proteins to obtain non-saturated images. Images were exported as .tif files using the Zeiss Axioversion 4.0.

### Flow cytometry analysis for apoptosis detection

Annexin-V/propidium iodide (PI) double assay was performed using the Annexin V, Alexa Fluor® 488 Conjugate Detection kit (Life technologies). Following doxorubicin treatment, cells were typsinized from culture dish and washed twice with cold PBS. 1 × 10^6^ cells were resuspended in 500 μL binding buffer and stained with 5 μL Alexa Fluor 488 conjugated annexin V according to the manufacturer’s instructions. 1 μL 100 μg/mL propidium iodide (PI) was mixed gently to cells for 15 min at room temperature in the dark. After incubation period, samples were subjected to FCM analysis in 1 h. using BD Accuri C6 Flow Cytometry (BD Biosciences, San Jose, CA). The data were analyzed using Accuri CFlow^@^ and CFlow Plus analysis software (BD Biosciences).

### Immunoblotting analysis

Immunoblotting analysis was used to validate the differential abundance of mass spectrometry identified proteins. The detailed experimental procedures were described in our previous reports [20-22]. All primary antibodies used for expression validation were purchased from Genetex (Hsinchu, Taiwan).

### 2D-DIGE, gel image analysis, protein staining, in-gel digestion and MALDI-TOF MS analysis

The detailed experimental procedures have been described in our previous publications [23-25]. Notably, peaks in the mass range of *m/z* 800-3000 were used to generate a peptide mass fingerprint that was searched against the Swiss-Prot/TrEMBL database (released on August 2011) with 531473 entries using Mascot software v2.3.02 (Matrix Science, London, UK). The parameters used for Mascot search are listed: *mouse*; tryptic digest with a maximum of 1 missed cleavage; carbamidomethylation of cysteine, partial protein N-terminal acetylation, partial methionine oxidation and partial modification of glutamine to pyroglutamate and a mass tolerance of 50 ppm. Identification was accepted based on significant MASCOT Mowse scores (*p* < 0.05), spectrum annotation and observed versus expected molecular weight and p*I* on 2-DE as well as at least 5 peptides in each identified protein.

## Results

### Quercetin facilitates cell survival and maintains cell morphology in doxorubicin-induced cell death in H9C2 cells

**Figure 1:**
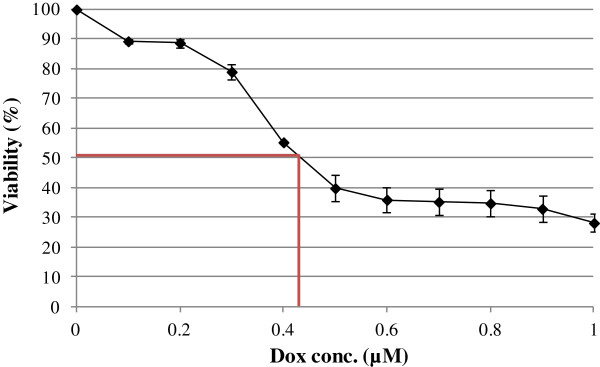
**Effect of doxorubicin treatment on H9C2 cell viability.** H9C2 cells were treated with indicated concentrations of doxorubicin from 3 independent experiments. Cell viability was determined by MTT assay after 24 h exposure of doxorubicin. Each data point indicates mean ± SD of triplicate values.

**Figure 2:**
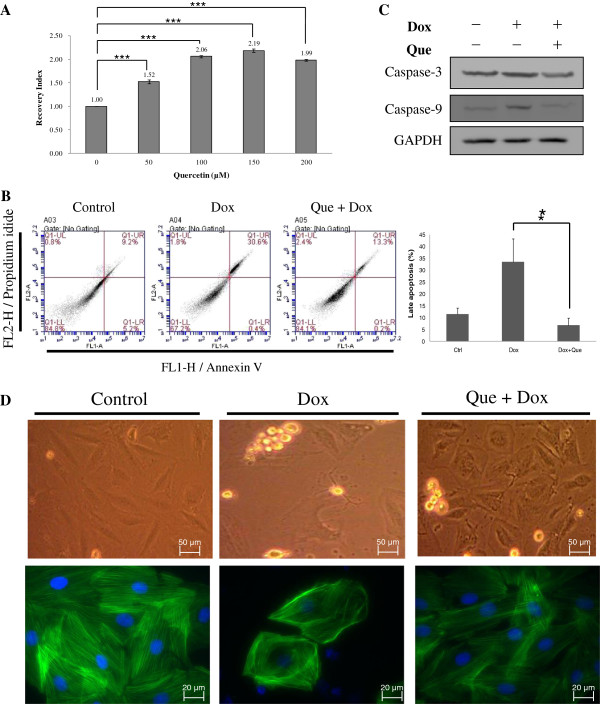
**Effects of quercetin on doxorubicin-induced changes of cell viability, cell apoptosis and cell morphology in H9C2 cells. (A)** MTT-based viability assays were performed on H9C2 cell cultures following treatments with different concentrations of quercetin (50 μM, 100 μM, 150 μM and 200 μM) or left untreated. Values were normalized against untreated samples and were the average of 4 independent measurements +/- the standard deviation. The statistic analysis was performed with two group paired Student t-test. **(B)** Typical dot plot diagrams detected annexin V-FITC and PI staining represent untreated, doxorubicin-treated, and quercetin-pretreated followed by doxorubicin–treated cells. The x-axis and y-axis stand for the intensity of annexin V-FITC and PI, respectively. The lower left area of presented background staining by annexin V-FITC and PI in normal cells, and apoptotic signals located in the right area. This figure is representative of 4 replicates. The statistic analysis of the replicates was listed in right panel. **(C)** The levels of caspase 3 and caspase 9 in H9C2 cells were detected by immunoblotting. GAPDH served as a sample loading control. **(D)** Cell morphology and protein location of F-actin in H9C2 cells were analyzed by immunostaining. H9C2 cells on coverslips were either left untreated, treated with doxorubicin or pre-treated with quercetin prior to doxorubicin treatment before fixation and staining. F-actin was stained with phalloidin and nuclei were stained with DAPI. Each set of five fields were taken using the same exposure and images are representative of five different fields. In **(B)** ~ **(D)**, H9C2 cells were untreated, 0.45 μM of doxorubicin for 24 h, or 100 μM of quercetin for 4 h followed by 0.45 μM of doxorubicin for 24 h.

To evaluate the effect of doxorubicin on rat cardiomyocytes (H9C2), we exposed the cells to doxorubicin in a range of 0-1 μM for 24 h in a serum-free medium. After exposure to doxorubicin, dose dependent loss of cell viabilities was observed in the H9C2 cells in 3 independent experiments using MTT assays (Figure 1). At a concentration of 0.45 μM, a significant loss (50%) of cell viability was detected after 24 h. To verify the role of quercetin regarding the recovery of doxorubicin-induced cardiomyopathy, we investigated the changes in cell viability in the H9C2 cells incubated in 0 μM, 50 μM, 100 μM, 150 μM and 200 μM quercetin for 4 h, followed by 24 h-exposure to 0.45 μM doxorubicin. Our results demonstrated that cell viability was significantly improved using quercetin in concentrations from 50 to 200 μM (Figure 2A).

Because excess ROS stress from doxorubicin-treated cardiomyocyte alters redox homeostasis and induces cell death, cell apoptosis was further detected using FACS. During cell apoptosis, phosphatidylserine is translocated to the outer surface of the plasma membrane, which has a high affinity to annexin V-FITC, and PI can penetrate the cell nucleus. As shown in Figure 2B, the apoptotic rate increased from 4.9% to 61.4% after doxorubicin treatment, whereas the apoptotic rate decreased to 9.5% after the H9C2 cells were pretreated with quercetin before doxorubicin treatment. In addition, the levels of the proteolytic enzymes, caspase 9 and caspase 3, were detected using immunoblotting in control, doxorubicin-treated and quercetin-pretreated H9C2 cells. Figure 2C indicates increased expression levels of the apoptosis factors for caspase 3 and caspase 9 after doxorubicin treatment. Quercetin protected the H9C2 cells from doxorubicin-induced cell injury by inhibiting the expressions of caspase 3 and caspase 9. Additionally, immunostained images of F-actin indicated that doxorubicin treatment affected cytoskeletal protein reorganization, causing cell morphology alternation (Figure 2D). Therefore, quercetin pretreatment is essential to maintaining doxorubicin-induced morphological changes.

### 2D-DIGE analysis of untreated and doxorubicin-treated H9C2 cells and quercetin pretreatment followed by doxorubicin treatment

**Figure 3:**
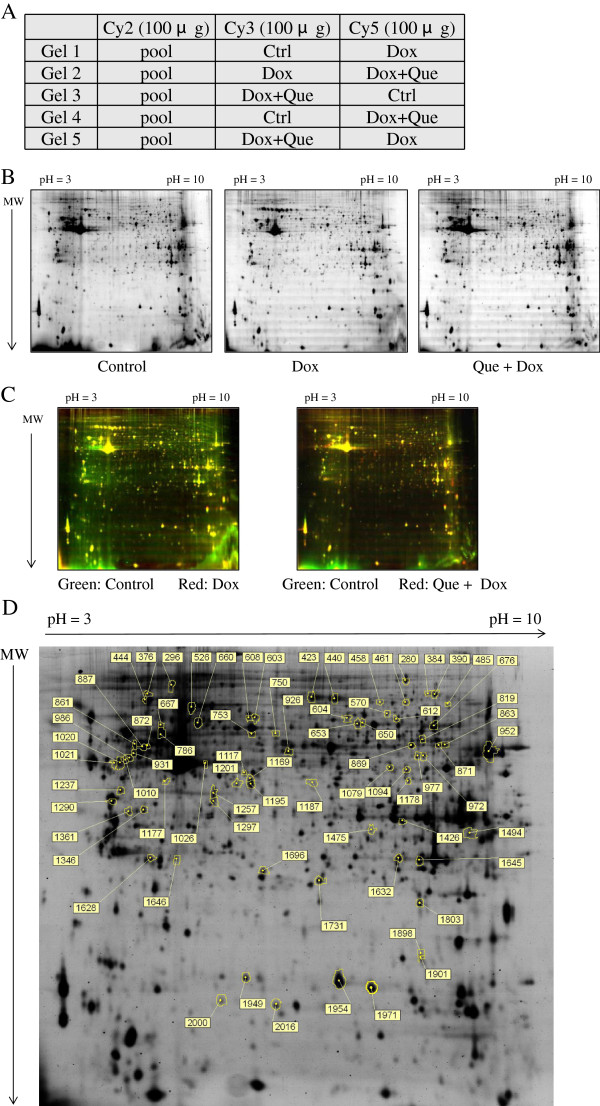
**2D-DIGE analysis of H9C2 cells in response to doxorubicin treatment and pre-treatment with quercetin. (A)** Samples arrangement for a triplicate 2D-DIGE experiment. **(B)** Protein samples (100 μg each) were labeled with Cy-dyes and separated using 24 cm, pH 3-10 non-linear IPG strips. 2D-DIGE images of the protein samples from H9C2 cells in response to doxorubicin treatment and pre-treatment with quercetin at appropriate excitation and emission wavelengths were shown as well as overlaid pseudo-colored images processed with ImageQuant Tool (GE Healthcare) **(C)**. **(D)** Protein samples (100 μg each) purified from total cell lysates were labeled with Cy-dyes and separated using 24 cm, pH 3-10 non-linear IPG strips. The differentially expressed protein features were annotated with spot numbers. In this 2D-DIGE experiment, H9C2 cells were untreated, 0.45 μM of doxorubicin for 24 h, or 100 μM of quercetin for 4 h followed by 0.45 μM of doxorubicin for 24 h.

**Figure 4:**
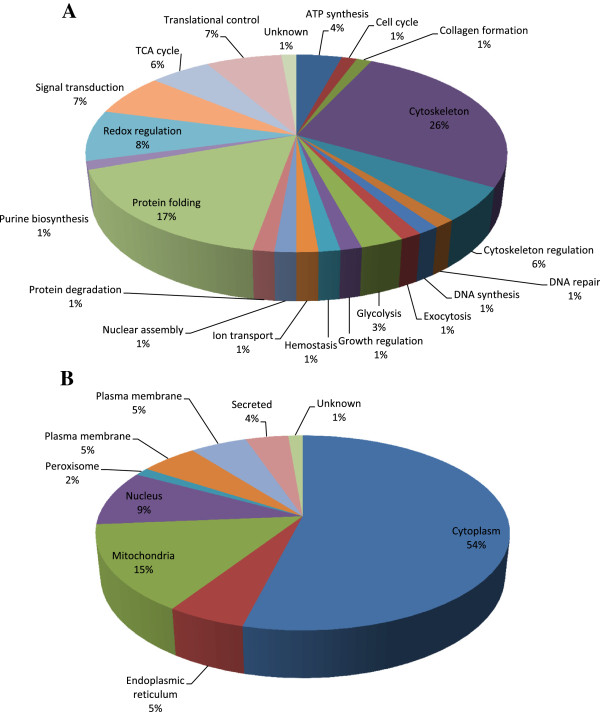
Percentage of total differentially expressed proteins identified by 2D-DIGE/MALDI-TOF MS for H9C2 cells in response to doxorubicin treatment and pre-treatment with quercetin according to their biological functions (A) and sub-cellular locations (B).

To fully understand the roles of doxorubicin and quercetin pretreatment in H9C2 cells, lysates of cells untreated, treated with doxorubicin, or treated with doxorubicin after pretreatment of quercetin, were subjected to 2D-DIGE analysis. The results of the 2D-DIGE analysis and DeCyder processing identified 2156 protein spots, and 73 proteins exhibited differential expression (≧ 1.5 fold or ≦-1.5 fold; *p* < 0.05) among the 3 conditions (Figure 3). Additional file 1: Table S1 shows the 73 proteins that were identified using MALDI-TOF MS, and 31 of the 73 identified protein spots that displayed doxorubicin-dependent alteration can be reversed by pretreating with quercetin (Additional file 1: Table S1 and Additional file 2). For example, the 78 kDa glucose-regulated protein (GRP-78) (No.444) was up-regulated (1.63 fold) in the doxorubicin-treated cells, whereas quercetin reduced the overexpression of doxorubicin-treated GRP-78 (-1.85 fold). The result suggested that the protective mechanisms of quercetin significantly altered the levels of chaperone proteins during doxorubicin-treatment in the cardiomyocytes. Figure 4A shows the functional distribution of the identified proteins from the 2D-DIGE results. Most of the proteins identified using MALDI-TOF MS were associated with the cytoskeletal element and cell migration as well as protein biosynthesis and metabolism, implying that quercetin is crucial for sustaining cytoskeletal and metabolic alternations responding to oxidative damage in the cardiomyocytes. During doxorubicin-mediated cardiomyopathy, the majority of the identified proteins were located in the cytoplasm and the nucleus (Figure 4B).

### Verifying the 2D-DIGE results by using immunoblotting and immunostaining

**Figure 5:**
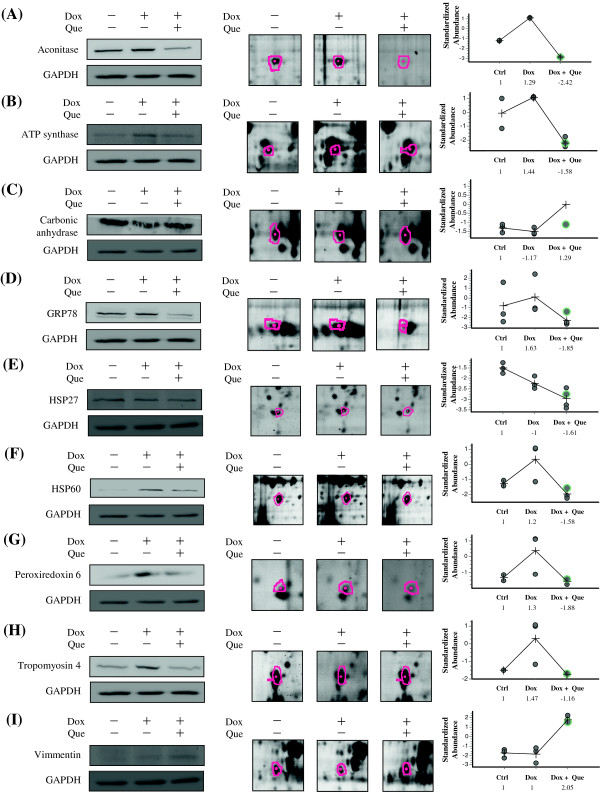
**Representative immunoblotting and immunofluorescent analyses for selected differentially expressed proteins identified by proteomic analysis of H9C2 cells in response to doxorubicin treatment and pre-treatment with quercetin.** The levels of identified proteins, **(A)** Aconitase, **(B)** ATP synthase, **(C)** Carbonic anhydrase, **(D)** GRP78, **(E)** HSP27, **(F)** HSP60, **(G)** Peroxiredoxin 6, **(H)** Tropomyosin 4, **(I)** Vimmentin, in H9C2 cells in response to doxorubicin treatment and pre-treatment with quercetin were confirmed by immunoblot, while GAPDH was used as loading controls (left panels). The protein expression maps and two-dimensional spot images were shown in right panels and middle panels, respectively. **(J)** H9C2 cells in different treatment conditions were fixed and incubated with anti-Cofilin-1 antibody (Red) and stained with Phalloidin (Green). Nucleus were stained with DAPI (Blue). Each set of three fields was taken using the same exposure, and images are representative of three different fields. In this validation experiment, H9C2 cells were left untreated, treated with 0.45 μM doxorubicin for 24 h or pretreated with 100 μM quercetin for 4 h followed by 0.45 μM doxorubicin for further 24 h.

**Figure 6:**
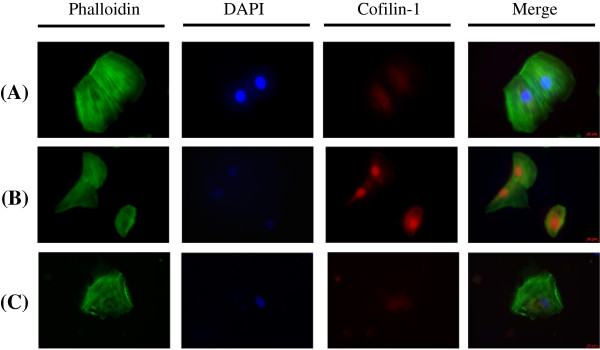
**Representative immunofluorescent analyses for Cofilin identified by proteomic analysis of H9C2 cells in response to doxorubicin treatment and pre-treatment with quercetin.**H9C2 cells in different treatment conditions were fixed and incubated with anti-Cofilin-1 antibody (Red) and stained with Phalloidin (Green). Nucleus was stained with DAPI (Blue). Each set of three fields was taken using the same exposure, and images are representative of three different fields. In this validation experiment, H9C2 cells were left untreated, treated with 0.45 µM doxorubicin for 24 h or pretreated with 100 µM quercetin for 4 h followed by 0.45 µM doxorubicin for further 24 h.

The levels of aconitase, ATP synthase, carbonic anhydrase, GRP78, HSP27, HSP60, peroxiredoxin 6, tropomyosin 4, vimmentin and cofilin-1 were examined using immunoblotting and immunostaining to validate the results of the 2D-DIGE analysis. The results indicated that aconitase, ATP synthase, GRP78, HSP60, peroxiredoxin 6, tropomyosin 4 and cofilin-1 were overexpressed in response to doxorubicin. However, quercetin suppressed the expression of the proteins during doxorubicin treatment in the H9C2 cells (Figure 5 and Figure 6). These results are consistent with the 2D-DIGE results.

## Discussion

Myocardial damage induced by doxorubicin was primarily caused by chelating DNA, inhibiting topoisomerase II and producing free radicals. Based on these concepts, numerous studies have evaluated the effects of doxorubicin-induced toxicity and the mechanisms that contribute to protecting cardiomyocytes [26-28]. In our previous studies, we reported on the cellular oxidative targets during heart damage induced by doxorubicin [29]. Additionally, we demonstrated that quercetin might dephosphorylate Src kinase activity in ROS-induced H9C2 cells and block ROS-induced inflammatory responses through STAT3 kinase. These activities contribute to preventing ischemia and reperfusion injury in cardiomyocytes [30]. In this study, we determined that quercetin treatment protected cardiomyocytes in the doxorubicin-induced heart damage model. Moreover, quercetin significantly facilitated cell survival by inhibiting cell apoptosis and maintaining cell morphology by inducing cytoskeletal protein rearrangement. Furthermore, after the proteomic analysis, we observed dramatic reductions in the H9C2 proteins involving protein folding, redox-regulation, and energy metabolism, as well as significant increases in proteins involving cytoskeleton and cytoskeleton regulatory proteins among the lysates of cells that were untreated, treated with doxorubicin, or treated with doxorubicin after pretreatment with quercetin. We suggest that cardiomyocytes develop defense mechanisms to overcome doxorubicin-induced ROS accumulation, which causes cell damage and cell death. The defense mechanisms include the overexpression of redox-modulated proteins to scavenge doxorubicin-induced ROS. In this study, pretreating with quercetin might scavenge ROS, diminishing oxidative stress and subsequently downregulating redox-regulatory proteins including glutamate dehydrogenase 1, isocitrate dehydrogenase, NADP-dependent malic enzyme, retinal dehydrogenase 1 and peroxiredoxin-6.

Moreover, we also observed that most proteins critical for modulating protein folding were down-regulated during pretreatment with quercetin which might account for the decreased concentrations of ROS causing the decrease of incorrectly folding proteins and attenuating the expression of chaperone proteins such as 60 kDa heat shock protein, 78 kDa glucose-regulated protein, alpha-crystallin B, heat shock protein beta-1, Stress-induced-phosphoprotein 1 and T-complex protein 1. Similar results have been reported in ethanol- and arsenite-induced oxidative stress, which caused over-expression of anti-oxidative stress proteins. However, the over-expression was significantly alleviated by quercetin pre-treatment [31,32]. In addition to these studies, the quercetin-induced downregulation of the protective proteins might account for the quercetin-treated cells exhibiting higher sensitivity to ROS damage, such as cancer cells [33,34]. Several key regulatory proteins mediated the interaction of heat shock proteins to inhibit apoptosis. The intrinsic pathway of caspase-mediated apoptosis was stimulated by c-Jun kinase, resulting in the release of cytochrome c from the mitochondria, and the subsequent activation of a caspase cascade involving caspase 8 and caspase 3. They were each inhibited by heat shock cognate 71, which interacted with Bcl-2 through Bag-1, enabling the complex to be incorporated into the mitochondrial membrane to inhibit apoptosis [35]. In this study, heat shock cognate 71 was upregulated in the quercetin-pretreated H9C2 cells, implying that heat shock cognate 71 is essential for protecting H9C2 cells from doxorubicin-induced apoptosis.

Quercetin was also observed to modulate the expression of cytoskeletal proteins (e.g., tubulins) and migration-regulated proteins (e.g., tubulin polymerization-promoting proteins) after encountering doxorubicin-induced damage [36]. Our immunofluorescence study demonstrated that quercetin can promote F-actin organization. Proteomic data also suggested that actin molecules were overexpressed during quercetin pretreatment, implying that quercetin causes the efficient regulation of protrusion dynamics and the wound healing of doxorubicin-damaged cardiomyocytes.

Our proteomic analysis indicated that quercetin pretreatment might down-regulate the levels of proteins involving energy metabolism including mitochondrial ATP synthesis, glycolytic proteins and TCA cycle proteins. Similar results were also reported by Dihal et al., who observed that glycolytic proteins were significantly down-regulated in their report. Additionally, Shoshan et al. reported that quercetin can modulate mitochondrial energy production by interacting with ATP synthase and blocking the enzyme’s activity [37,38]. The current proteomic analysis corresponded with these results.

Our preliminary data indicated that quercetin reduces but enhances the cytotoxicity of doxorubicin on cardiomyocyte H9C2 cells and liver cancer HepG2 cells, respectively (data not shown). This observation suggested the potential of combining quercetin and doxorubicin for treating liver cancer. Although no direct evidence indicates the cooperative effect of quercetin and doxorubicin on other cancer treatment, performing relevant evaluations of other cancers is worthwhile in the future.

In summary, this study is the first to report on the principle mechanism of quercetin against doxorubicin-induced cytotoxicity in cardiomyocytes, using cell biology and a quantitative proteomic analysis. The information obtained in this study presents the potential of combining quercetin with doxorubicin to achieve reduced cardiotoxicity in cancer chemotherapy.

## Conclusions

This study is the first to report detailed protective mechanisms for the action of quercetin against doxorubicin-induced cardiomyocyte toxicity. Quercetin might stimulate cardiomyocytes to repair damage after treating doxorubicin by modulating metabolic activation, protein folding and cytoskeleton rearrangement.

## Abbreviations

1-DE: One-dimensional gel electrophoresis; 2-DE: Two-dimensional gel electrophoresis; Ab: Antibody; ddH2O: double deionized water; DIGE: Differential gel electrophoresis; DTT: Dithiothreitol; FCS: Fetal calf serum; MALDI-TOF MS: Matrix assisted laser desorption ionization-time of flight mass spectrometry.

## Competing interests

The authors declare that they have no competing interests.

## Authors’ contributions

HCC designed the experiments and wrote the drafting manuscript. JYC, RYH performed the cell culture, 2D-gel electrophoresis, image analysis, cell biological analysis and immunoblotting. HCC supervised the experiments and the data analysis and finalized the manuscript. All authors have read and approved the final manuscript.

## Supplementary Material

Additional file 1: Table S1 Differentially expressed proteins were listed alphabetically after 2D-DIGE and MALDI-TOF Mass spectrometry analysis in H9C2 cells in response to doxorubicin treatment and quercetin pretreatment. The average ratios of these 73 spots are differentially expressed among untreated (control), doxorubicin-treated and quercetin-pretreated followed by doxorubicin-treated cells, calculated from triplicate gels. Click here for file

Additional file 2 **Raw spectra of the identified proteins from Additional file ****1****: Table S1** Click here for file

## References

[B1] Verma S Dent S Chow BJ Rayson D Safra T Metastatic breast cancer: the role of pegylated liposomal doxorubicin after conventional anthracyclines Cancer Treat Rev 2008 20 391 406 10.1016/j.ctrv.2008.01.008 18358614

[B2] Vatsyayan R Chaudhary P Lelsani PC Singhal P Awasthi YC Awasthi S Singhal SS Role of RLIP76 in doxorubicin resistance in lung cancer (Review) Int J Oncol 2009 20 1505 1511 19424567 10.3892/ijo_00000279 PMC2916676

[B3] Green AE Rose PG Pegylated liposomal doxorubicin in ovarian cancer Int J Nanomedicine 2006 20 229 239 17717964 PMC2426807

[B4] Christiansen S Autschbach R Doxorubicin in experimental and clinical heart failure Eur J Cardiothorac Surg 2006 20 611 616 10.1016/j.ejcts.2006.06.024 16901709

[B5] Kalishina EV Saprin AN Solomka VS Shchebrak NP Piruzian LA Inhibition of hydrogen peroxide, oxygen and semiquinone radicals in the development of drug resistance to doxorubicin in human erythroleukemia K562-cells Vopr Onkol 2003 20 294 298 12926210

[B6] Simunek T Sterba M Popelova O Adamcova M Hrdina R Gersl V Anthracycline-induced cardiotoxicity: overview of studies examining the roles of oxidative stress and free cellular iron Pharmacol Rep 2009 20 154 171 19307704 10.1016/s1734-1140(09)70018-0

[B7] Joshi G Aluise CD Cole MP Sultana R Pierce WM Vore M St Clair DK Butterfield DA Alterations in brain antioxidant enzymes and redox proteomic identification of oxidized brain proteins induced by the anti-cancer drug adriamycin: implications for oxidative stress-mediated chemobrain Neuroscience 2010 20 796 807 10.1016/j.neuroscience.2010.01.021 20096337 PMC2852883

[B8] Keenan J Murphy L Henry M Meleady P Clynes M Proteomic analysis of multidrug-resistance mechanisms in adriamycin-resistant variants of DLKP, a squamous lung cancer cell line Proteomics 2009 20 1556 1566 10.1002/pmic.200800633 19242932

[B9] Venkatakrishnan CD Tewari AK Moldovan L Cardounel AJ Zweier JL Kuppusamy P Ilangovan G Heat shock protects cardiac cells from doxorubicin-induced toxicity by activating p38 MAPK and phosphorylation of small heat shock protein 27 Am J Physiol Heart Circ Physiol 2006 20 H2680 H2691 10.1152/ajpheart.00395.2006 16782845

[B10] Boots AW Haenen GR Bast A Health effects of quercetin: from antioxidant to nutraceutical Eur J Pharmacol 2008 20 325 337 10.1016/j.ejphar.2008.03.008 18417116

[B11] Sanhueza J Valdes J Campos R Garrido A Valenzuela A Changes in the xanthine dehydrogenase/xanthine oxidase ratio in the rat kidney subjected to ischemia-reperfusion stress: preventive effect of some flavonoids Res Commun Chem Pathol Pharmacol 1992 20 211 218 1475527

[B12] Sun B Sun GB Xiao J Chen RC Wang X Wu Y Cao L Yang ZH Sun XB Isorhamnetin inhibits H (2) O (2)-induced activation of the intrinsic apoptotic pathway in H9c2 cardiomyocytes through scavenging reactive oxygen species and ERK inactivation J Cell Biochem 2012 20 473 485 10.1002/jcb.23371 21948481

[B13] Kyaw M Yoshizumi M Tsuchiya K Kirima K Tamaki T Antioxidants inhibit JNK and p38 MAPK activation but not ERK 1/2 activation by angiotensin II in rat aortic smooth muscle cells Hypertens Res 2001 20 251 261 10.1291/hypres.24.251 11409648

[B14] Staedler D Idrizi E Kenzaoui BH Juillerat-Jeanneret L Drug combinations with quercetin: doxorubicin plus quercetin in human breast cancer cells Cancer Chemother Pharmacol 2011 20 1161 1172 10.1007/s00280-011-1596-x 21400027

[B15] Kaiserova H Simunek T van der Vijgh WJ Bast A Kvasnickova E Flavonoids as protectors against doxorubicin cardiotoxicity: role of iron chelation, antioxidant activity and inhibition of carbonyl reductase Biochim Biophys Acta 2007 20 1065 1074 10.1016/j.bbadis.2007.05.002 17572073

[B16] Zhou J Liang S Fang L Chen L Tang M Xu Y Fu A Yang J Wei Y Quantitative proteomic analysis of HepG2 cells treated with quercetin suggests IQGAP1 involved in quercetin-induced regulation of cell proliferation and migration OMICS 2009 20 93 103 10.1089/omi.2008.0075 19207037

[B17] Aalinkeel R Bindukumar B Reynolds JL Sykes DE Mahajan SD Chadha KC Schwartz SA The dietary bioflavonoid, quercetin, selectively induces apoptosis of prostate cancer cells by down-regulating the expression of heat shock protein 90 Prostate 2008 20 1773 1789 10.1002/pros.20845 18726985 PMC2826114

[B18] Timms JF Cramer R Difference gel electrophoresis Proteomics 2008 20 4886 4897 10.1002/pmic.200800298 19003860

[B19] Lin ST Chou HC Chang SJ Chen YW Lyu PC Wang WC Chang MD Chan HL Proteomic analysis of proteins responsible for the development of doxorubicin resistance in human uterine cancer cells J Proteomics 2012 20 5822 5847 10.1016/j.jprot.2012.07.047 22889595

[B20] Hung PH Chen YW Cheng KC Chou HC Lyu PC Lu YC Lee YR Wu CT Chan HL Plasma proteomic analysis of the critical limb ischemia markers in diabetic patients with hemodialysis Mol Biosyst 2011 20 1990 1998 10.1039/c1mb05055a 21468429

[B21] Lin CP Chen YW Liu WH Chou HC Chang YP Lin ST Li JM Jian SF Lee YR Chan HL Proteomic identification of plasma biomarkers in uterine leiomyoma Mol Biosyst 2011 20 1136 1145 22193648 10.1039/c2mb05453a

[B22] Chen YW Liu JY Lin ST Li JM Huang SH Chen JY Wu JY Kuo CC Wu CL Lu YC Proteomic analysis of gemcitabine-induced drug resistance in pancreatic cancer cells Mol Biosyst 2011 20 3065 3074 10.1039/c1mb05125c 21894339

[B23] Wu CL Chou HC Cheng CS Li JM Lin ST Chen YW Chan HL Proteomic analysis of UVB-induced protein expression- and redox-dependent changes in skin fibroblasts using lysine- and cysteine-labeling two-dimensional difference gel electrophoresis J Proteomics 2012 20 1991 2014 10.1016/j.jprot.2011.12.038 22270008

[B24] Lai TC Chou HC Chen YW Lee TR Chan HT Shen HH Lee WT Lin ST Lu YC Wu CL Secretomic and proteomic analysis of potential breast cancer markers by two-dimensional differential gel electrophoresis J Proteome Res 2010 20 1302 1322 10.1021/pr900825t 20052998

[B25] Chou HC Lu YC Cheng CS Chen YW Lyu PC Lin CW Timms JF Chan HL Proteomic and redox-proteomic analysis of berberine-induced cytotoxicity in breast cancer cells J Proteomics 2012 20 3158 3176 10.1016/j.jprot.2012.03.010 22522123

[B26] Mokni M Hamlaoui-Guesmi S Amri M Marzouki L Limam F Aouani E Grape seed and skin extract protects against acute chemotherapy toxicity induced by doxorubicin in rat heart Cardiovasc Toxicol 2012 20 158 165 10.1007/s12012-012-9155-1 22290400

[B27] Calvo-Romero JM Fernandez-Soria-Pantoja R Arrebola-Garcia JD Gil-Cubero M Ischemic heart disease associated with vincristine and doxorubicin chemotherapy Ann Pharmacother 2001 20 1403 1405 11724093 10.1345/aph.10358

[B28] Chen JY Chan HL Chou HC Proteomic analysis of quercetin-induced cardioprotective effects Genomic Med, Biomarkers, and Health Sci 2012 20 51 53 10.1016/j.gmbhs.2012.04.006

[B29] Lin ST Chou HC Chen YW Chan HL Redox-proteomic analysis of doxorubicin-induced altered thiol activity in cardiomyocytes Mol Biosyst 2013 20 447 456 10.1039/c2mb25367d 23340498

[B30] Chen YW Chou HC Lin ST Chen YH Chang YJ Chen L Chan HL Cardioprotective effects of quercetin in cardiomyocyte under ischemia/reperfusion injury Evid Based Complement Alternat Med 2013 20 364519 23573126 10.1155/2013/364519 PMC3612448

[B31] Oliva J Bardag-Gorce F Tillman B French SW Protective effect of quercetin, EGCG, catechin and betaine against oxidative stress induced by ethanol in vitro Exp Mol Pathol 2011 20 295 299 10.1016/j.yexmp.2011.02.006 21352821 PMC3113678

[B32] Bongiovanni GA Soria EA Eynard AR Effects of the plant flavonoids silymarin and quercetin on arsenite-induced oxidative stress in CHO-K1 cells Food Chem Toxicol 2007 20 971 976 10.1016/j.fct.2006.12.002 17240505

[B33] Gibellini L Pinti M Nasi M De Biasi S Roat E Bertoncelli L Cossarizza A Interfering with ROS metabolism in cancer cells: the potential role of Quercetin Cancers (Basel) 2010 20 1288 1311 10.3390/cancers2021288 24281116 PMC3835130

[B34] Tytell M Hooper PL Heat shock proteins: new keys to the development of cytoprotective therapies Expert Opin Ther Targets 2001 20 267 287 10.1517/14728222.5.2.267 15992180

[B35] Takayama S Krajewski S Krajewska M Kitada S Zapata JM Kochel K Knee D Scudiero D Tudor G Miller GJ Expression and location of Hsp70/Hsc-binding anti-apoptotic protein BAG-1 and its variants in normal tissues and tumor cell lines Cancer Res 1998 20 3116 3131 9679980

[B36] Zhang X Xu Q Saiki I Quercetin inhibits the invasion and mobility of murine melanoma B16-BL6 cells through inducing apoptosis via decreasing Bcl-2 expression Clin Exp Metastasis 2000 20 415 421 10.1023/A:1010960615370 11467774

[B37] Dihal AA der WH V Hendriksen PJ Charif H Dekker LJ Ijsselstijn L De Boer VC Alink GM Burgers PC Rietjens IM Transcriptome and proteome profiling of colon mucosa from quercetin fed F344 rats point to tumor preventive mechanisms, increased mitochondrial fatty acid degradation and decreased glycolysis Proteomics 2008 20 45 61 10.1002/pmic.200700364 18095365

[B38] Shoshan V Shahak Y Shavit N Quercetin interaction with the chloroplast ATPase complex Biochim Biophys Acta 1980 20 421 433 10.1016/0005-2728(80)90173-5 6446936

