# Supplementary material for: Quercetin-induced cardioprotection against doxorubicin cytotoxicity
Source: J Biomed Sci. 2013 Dec 20;20(1):95. doi: 10.1186/1423-0127-20-95 (PMC3898810; doi:10.1186/1423-0127-20-95)
Supplement: Additional file 1: Table S1 — Differentially expressed proteins were listed alphabetically after 2D-DIGE and MALDI-TOF Mass spectrometry analysis in H9C2 cells in response to doxorubicin treatment and quercetin pretreatment. The average ratios of these 73 spots are differentially expressed among untreated (control), doxorubicin-treated and quercetin-pretreated followed by doxorubicin-treated cells, calculated from triplicate gels. [file 1423-0127-20-95-S1.pdf]

Table S1. Differentially expressed proteins were listed alphabetically after 2D-DIGE and MALDI-TOF Mass spectrometry analysis in H9C2 cells in response to doxorubicin treated and pre-treated with querc

No.	Swissprot No.	Protein name	Theoretical MW	Theoretical pI	Observed MW	Observed pI	No. Match. Peptides	Cov. (%)	MOWSE Score	Functional classification	Subcellular location	IC50 / Ctrl	t-test	Que / Ctrl	t-test	Que / IC50	t-test
977	P62198	26S protease regulatory subunit 8	45768	7.11	50000	8.5	6/25	16	65/56	Protein degradation	Cytoplasm	-1.1	0.33	-1.54	0.012	-1.4	0.041
660	P63039	60 kDa heat shock protein, mitochondrial/HSP-60	61088	5.91	61000	6.0	10/17	13	108/56	Protein folding	Mitochondria	1.23	0.22	-1.58	0.075	-1.94	0.007
376	P06761	78 kDa glucose-regulated protein/GRP-78	72473	5.07	75000	5.0	12/27	21	106/56	Protein folding	Endoplasmic reticulum	1.41	0.08	1.79	0.022	1.27	0.2
444	P06761	78 kDa glucose-regulated protein/GRP-78	72473	5.07	72000	4.9	10/20	17	98/56	Protein folding	Endoplasmic reticulum	1.63	0.35	-1.85	0.26	-3.02	0.026
1178	Q5XI22	Acetyl-CoA acetyltransferase, cytosolic	41538	6.86	45000	8.3	7/14	19	94/56	TCA cycle	Mitochondria	-1.61	0.0047	-2.61	0.00064	-1.62	0.018
384	Q9ER34	Aconitate hydratase, mitochondrial/Aconitase	86121	7.87	80000	8.7	7/10	11	108/56	TCA cycle	Mitochondrion	1.25	0.15	-1.58	0.053	-1.97	0.018
390	Q9ER34	Aconitate hydratase, mitochondrial/Aconitase	86121	7.87	80000	8.8	8/18	13	103/56	TCA cycle	Mitochondrion	1.29	0.0025	-2.42	2.20E-06	-3.13	7.60E-07
1021	P60711	Actin, cytoplasmic 1	42052	5.29	49000	4.0	6/18	18	102/56	Cytoskeleton	Cytoplasm	1.26	0.11	2.1	0.0034	1.67	0.0088
1117	P60711	Actin, cytoplasmic 1	42052	5.29	48000	6.5	5/10	16	80/56	Cytoskeleton	Cytoplasm	-1.23	0.15	1.87	0.0092	2.31	0.0022
1297	P60711	Actin, cytoplasmic 1	42052	5.29	43000	6.3	5/10	18	82/56	Cytoskeleton	Cytoplasm	1.03	0.69	2.15	0.002	2.08	0.0033
1361	P60711	Actin, cytoplasmic 1	42052	5.29	42000	4.5	5/10	15	80/56	Cytoskeleton	Cytoplasm	1.03	0.78	2.05	0.00054	1.98	0.00056
1646	P60711	Actin, cytoplasmic 1	42052	5.29	30000	5.5	5/10	16	66/56	Cytoskeleton	Cytoplasm	1.14	0.4	1.6	3.60E-05	1.41	0.019
1026	P60711	Actin, cytoplasmic 1	42052	5.29	49000	6.1	9/19	26	81/56	Cytoskeleton	Cytoplasm	1.16	0.21	1.63	0.029	1.4	0.093
1898	P23928	Alpha-crystallin B chain	20076	6.76	20000	8.5	6/22	25	72/56	Protein folding	Cytoplasm	1.38	0.03	-1.19	0.28	-1.64	0.011
1901	P23928	Alpha-crystallin B chain	20076	6.76	20000	8.5	5/14	25	75/56	Protein folding	Cytoplasm	1.19	0.29	-1.41	0.13	-1.68	0.021
1475	Q07936	Annexin A2	38939	7.55	40000	7.8	7/13	23	101/56	Signal transduction	Secreted	1.02	0.89	1.54	0.04	1.5	0.031
863	P15999	ATP synthase subunit alpha, mitochondrial	59831	9.22	55000	8.6	5/11	15	82/56	ATP synthesis	Mitochondria	1.16	0.24	-2.03	0.0046	-2.35	0.0018
871	P15999	ATP synthase subunit alpha, mitochondrial	59831	9.22	52000	8.8	8/16	16	119/56	ATP synthesis	Mitochondria	1.44	0.024	-1.58	0.003	-2.28	3.20E-05
887	P10719	ATP synthase subunit beta, mitochondrial	56318	5.19	52000	4.9	6/13	13	74/56	ATP synthesis	Mitochondria	1.13	0.26	-1.62	0.0068	-1.84	0.0033
612	O35567	Bifunctional purine biosynthesis protein PURH/IMP	64681	6.69	65000	8.0	8/21	15	103/56	Purine biosynthesis	Mitochondria	-1.05	0.7	-1.73	0.0038	-1.65	0.0045

485	P14141	Carbonic anhydrase 3	29698	6.98	70000	9.1	6/18	32	83/56	Hemostasis	Cytoplasm	-1.18	0.37	-1.53	0.042	-1.3	0.047
1645	P14141	Carbonic anhydrase 3	29698	6.89	30000	8.5	5/12	28	93/56	Hemostasis	Cytoplasm	-1.17	0.29	1.29	0.078	1.5	0.01
1954	P45592	Cofilin-1	18749	8.22	18000	7.4	7/12	31	83/56	Cytoskeleton regulation	Cytoplasm	1.33	0.0039	-1.21	0.03	-1.62	0.00096
1971	Q7M0E3	Destrin	18807	8.19	18000	7.8	5/13	23	66/56	Cytoskeleton regulation	Cytoplasm	1.51	0.0023	-1.09	0.2	-1.64	0.0012
819	P97679	DNA mismatch repair protein Mlh1	85171	5.63	55000	8.8	7/14	7	68/56	DNA repair	Nucleus	1.46	0.006	-1.66	0.0045	-2.42	0.0004
972	P63036	DnaJ homolog subfamily A member 1	45581	6.65	50000	8.6	7/12	14	69/56	Protein folding	Mitochondria	-1.72	0.34	-3.39	0.023	-1.97	0.0082
280	P05197	Elongation factor 2/EF-2	96192	6.41	90000	8.2	8/10	8	85/56	Translational control	Cytoplasm	-1.07	0.59	-1.81	0.0042	-1.68	0.0032
461	P09759	Ephrin type-B receptor 1	111352	6.03	72000	8.2	6/15	7	58/56	Signal transduction	Plasma membrane	-1.09	0.22	-2.32	0.00039	-2.13	0.00064
1257	B0BNA7	Eukaryotic translation initiation factor 3 subunit I/eIF3i	36837	5.38	44000	6.1	6/17	23	94/56	Translational control	Cytoplasm	-1.07	0.61	1.74	0.036	1.87	0.02
1020	O54924	Exocyst complex component 8	81676	5.3	48000	4.2	8/13	13	72/56	Exocytosis	Cytoplasm	1.12	0.52	1.96	0.0033	1.75	0.0031
952	P31977	Ezrin	69462	5.83	50000	10.5	9/16	11	69/56	Cytoskeleton	Cytoplasm	-1.15	0.38	-1.72	0.0023	-1.5	0.026
869	P10860	Glutamate dehydrogenase 1, mitochondrial/GDH 1	61719	8.08	55000	8.3	6/10	12	88/56	Redox regulation	Mitochondria	1.37	0.0026	-1.39	0.00034	-1.91	5.00E-05
1803	P62828	GTP-binding nuclear protein Ran	24579	7.07	23000	8.4	5/11	26	91/56	Cell cycle	Nucleus	-1.32	0.012	-2.63	4.30E-05	-2	6.40E-05
1628	P63018	Heat shock cognate 71 kDa protein	71055	5.37	30000	5.0	6/17	14	85/56	Protein folding	Cytoplasm	1.73	0.038	2.69	0.004	1.56	0.062
1731	P42930	Heat shock protein beta-1	22936	6.2	25000	7.2	6/17	33	97/56	Protein folding	Cytoplasm	-1.49	0.036	-1.97	0.0028	-1.32	0.057
1177	Q07523	Hydroxyacid oxidase 2/HAOX2	39633	7.55	45000	5.4	5/20	17	62/56	Redox regulation	Peroxisome	1.01	0.97	1.63	0.01	1.62	0.1
1201	Q99NA5	Isocitrate dehydrogenase [NAD] subunit alpha, mitochondrial	40044	6.47	44000	6.4	7/24	17	80/56	TCA cycle	Mitochondria	1.33	0.03	1.78	0.0031	1.34	0.011
1079	P41562	Isocitrate dehydrogenase [NADP] cytoplasmic/IDH	47047	6.53	47000	8.0	5/16	11	60/56	Redox regulation	Cytoplasm	1.26	0.01	-1.51	0.0011	-1.9	0.0002
458	P48679	Lamin-A	74564	6.54	72000	7.9	7/20	11	75/56	Nuclear assembly	Nucleus	1.31	0.079	-1.54	0.093	-2.01	0.022
570	P48679	Lamin-A	74564	6.54	68000	7.9	10/23	15	125/56	Nuclear assembly	Nucleus	-1.05	0.59	-1.64	0.0011	-1.56	0.0048
296	Q6AXU7	MIF4G domain-containing protein	25854	5.03	80000	5.4	5/16	22	65/56	Translational control	Nucleus	1.12	0.33	-1.6	0.00031	-1.79	0.00077
786	P63086	Mitogen-activated protein kinase 1/MAP kinase 1	41648	6.5	58000	5.3	6/20	21	61/56	Signal transduction	Cytoplasm	1.01	0.86	-2.01	0.00097	-2.03	0.00048

1494	O35763	Moesin	67868	6.16	30000	9.8	6/8	7	63/56	Cytoskeleton	Plasma membrane	1.32	0.065	1.63	0.0053	1.24	0.0094
650	P13697	NADP-dependent malic enzyme/NADP-ME	64589	6.49	62000	7.6	5/13	9	71/56	Redox regulation	Cytoplasm.	-1.04	0.5	-1.59	0.0022	-1.54	0.0033
2016	Q05982	Nucleoside diphosphate kinase A/NDK A	17296	5.69	17000	6.8	4/11	31	76/56	DNA synthesis	Nucleus	1.16	0.0016	-1.32	0.21	-1.53	0.037
1426	P52944	PDZ and LIM domain protein 1	36018	6.79	40000	8.1	6/17	25	94/56	Cytoskeleton regulation	Cytoplasm	1.52	0.003	1.12	0.13	-1.36	0.0073
1696	O35244	Peroxiredoxin-6	24860	5.64	26000	6.6	6/12	29	90/56	Redox regulation	Cytoplasm	1.33	0.12	-1.18	0.34	-1.57	0.0084
1632	P25113	Phosphoglycerate mutase 1	28928	6.67	30000	8.1	6/21	35	95/56	Glycolysis	Cytoplasm	1.07	0.63	-1.52	0.0067	-1.63	0.0041
440	Q9ERS1	Potassium channel subfamily K member 12	48075	9.83	72000	7.4	6/12	15	60/56	Ion transport	Plasma membrane	1.25	NA	-1.52	NA	-1.9	0.007
608	P54001	Prolyl 4-hydroxylase subunit alpha-1/4-PH alpha-1	61202	5.63	62000	6.5	5/17	10	58/56	Collagen formation	Endoplasmic reticulum	-1.24	0.026	-1.63	0.0094	-1.32	0.049
1346	O35569	Pro-neuregulin-2, membrane-bound isoform/Pro-NRG2	95258	9.52	42000	5.0	8/17	7	65/56	Signal transduction	Secreted	1.04	0.7	-1.49	0.015	-1.55	0.014
1187	Q63081	Protein disulfide-isomerase A6/PDIA6	48542	5	45000	7.1	5/14	15	81/56	Protein folding	Endoplasmic reticulum	-1.01	0.99	1.64	0.083	1.67	0.0051
423	Q562C7	Pumilio domain-containing protein KIAA0020 homolog	73196	9.7	72000	7.1	7/17	12	66/56	Translational control	Nucleus	1.56	0.0046	-1.06	0.34	-1.66	0.0021
676	P11980	Pyruvate kinase isozymes M1/M2	58294	6.63	60000	8.8	7/24	17	84/56	Glycolysis	Cytoplasm	1.14	0.13	-1.38	0.009	-1.57	0.0028
926	P50399	Rab GDP dissociation inhibitor beta/Rab GDI beta	51018	5.93	50000	6.9	9/14	17	89/56	Signal transduction	Plasma membrane	-1.14	0.67	1.51	0.12	1.73	0.036
1094	P51647	Retinal dehydrogenase 1/RALDH 1	54994	7.94	46000	8.0	5/15	10	58/56	Redox regulation	Cytoplasm	-1.02	0.91	-1.68	0.0088	-1.65	0.0065
1237	P16975	SPARC	35129	4.81	44000	4.4	6/13	20	81/56	Growth regulation	Secreted	-2.12	0.0047	-2.62	0.00017	-1.24	0.12
653	O35814	Stress-induced-phosphoprotein 1/STI1	63158	6.4	60000	7.6	9/19	13	70/56	Protein folding	Cytoplasm	-1.13	0.17	-1.54	0.00061	-1.36	0.0013
2000	Q4QQU6	Survival of motor neuron-related-splicing factor 30	26938	6.78	18000	6.3	6/11	21	62/56	Translational control	Nucleus	1.57	0.008	1.14	0.11	-1.38	0.0033
604	Q6P502	T-complex protein 1 subunit gamma/TCP-1-gamma	61179	6.23	62000	7.5	14/23	25	134/56	Protein folding	Cytoplasm	-1.06	0.34	-1.53	0.0009	-1.45	0.00054
1290	P09495	Tropomyosin alpha-4 chain/Tropomyosin-4	28549	4.66	43000	4.0	8/20	21	68/56	Cytoskeleton	Cytoplasm	1.47	NA	-1.16	NA	-1.71	0.0088
750	Q6AYZ1	Tubulin alpha-1C chain	50590	4.96	58000	6.8	7/10	19	98/56	Cytoskeleton	Cytoplasm	1.29	0.18	1.79	0.019	1.38	0.049
753	Q5XIF6	Tubulin alpha-4A chain	50634	4.95	58000	6.5	4/14	14	56/56	Cytoskeleton	Cytoplasm	-1.36	0.026	-1.54	0.0065	-1.13	0.16
861	P69897	Tubulin beta-5 chain	50095	4.78	52000	4.6	8/10	16	87/56	Cytoskeleton	Cytoplasm	-1.19	0.17	-1.6	0.0085	-1.35	0.054
872	P69897	Tubulin beta-5 chain	50095	4.78	52000	5.0	7/19	15	83/56	Cytoskeleton	Cytoplasm	1.07	0.24	-1.44	0.02	-1.54	0.0092

1169	P69897	Tubulin beta-5 chain	50095	4.78	45000	6.5	5/23	13	76/56	Cytoskeleton	Cytoplasm	1.6	0.061	2.83	0.027	1.77	0.025
1195	P69897	Tubulin beta-5 chain	50095	4.78	44000	6.5	12/20	27	138/56	Cytoskeleton	Cytoplasm	1.25	0.054	2.2	0.00083	1.75	0.0022
603	Q5PPN5	Tubulin polymerization-promoting protein family member 3	19139	9.18	62000	6.6	7/27	38	89/56	Cytoskeleton regulation	Cytoplasm	-1.26	0.0017	-2	0.0017	-1.59	0.01
1949	Q6AY88	Uncharacterized protein C1orf172 homolog	44273	6.04	18000	6.4	6/17	13	58/56	Unknown	Unknown	-1.13	0.29	-1.51	0.022	-1.33	0.02
667	P31000	Vimentin	53757	5.06	60000	5.2	13/38	28	146/56	Cytoskeleton	Cytoplasm	1.07	0.46	-1.43	0.083	-1.53	0.026
931	P31000	Vimentin	53757	5.06	50000	4.5	9/15	22	101/56	Cytoskeleton	Cytoplasm	1.51	0.18	2.31	0.0057	1.53	0.024
986	P31000	Vimentin	53757	5.06	48000	4.5	11/18	19	106/56	Cytoskeleton	Cytoplasm	1	0.97	2.05	0.0078	2.04	0.014
1010	P31000	Vimentin	53757	5.06	48000	4.4	9/29	18	96/56	Cytoskeleton	Cytoplasm	1.03	0.97	3.02	0.00098	2.93	0.0054
